# Supplementary material for: A genetic locus complements resistance to Bordetella pertussis-induced histamine sensitization
Source: Commun Biol. 2023 Mar 6;6:244. doi: 10.1038/s42003-023-04603-w (PMC9988836; doi:10.1038/s42003-023-04603-w)

Supplementary Table 1. List of 50 inbred mouse strains used in genetic association testing.

Bphs Susceptible (41)				Bphs Resistant (9)
129S1/SvImJ	C57BL/6J	LP/J	PL/J	AKR/J
129T2/SvEmsJ	C57BLKS/J	MA/MyJ	PWD/PhJ	C3H/HeJ
129X1/SvJ	C57BR/cdJ	MOLD/RkJ	PWK/PhJ	CAST/EiJ
A/J	C57L/J	MOLF/EiJ	RF/J	CBA/J
A/WySnJ	C58/J	NOD/ShiLtJ	RIIIS/J	I/LnJ
BALB/cJ	CZECHII/EiJ	NON/ShiLtJ	SJL/J	MRL/MpJ
BALB/cByJ	DBA/1J	NOR/LtJ	SM/J	MSM/MsJ
BPL/1J	DBA/2J	NU/J	SWR/J	SF/CamEiJ
C57BL/10J	FVB/NJ	NZB/BlNJ		SKIVE/EiJ
C57BL/10ScNJ	JF1/MsJ	NZW/LacJ		
C57BL/6ByJ	LG/J	P/J		

Supplementary Table 2. Genetic association analysis identifies variants on Chr6 associated with Bphs^{s}.

Chr	bp (mm10)	SNP	Gene	*p*-value$_{EMMA}$
6	114780885	rs31440528	*I:Atg7*	8.10E-07
6	114732069	rs31439505	*I:Atg7*	2.39E-06
6	114758979	rs31445205	*I:Atg7*	2.39E-06
**Suggestive SNPs (p<0.05)**				
6	110966239	rs30498521	*I:Grm7*	1.07E-03
6	113466213	rs37366170	*I:Il17re*	3.14E-03
6	113521759	rs36681994	*I:Emc3*	3.14E-03
6	113766702	rs36921136	*I:Atp2b2*	3.14E-03
6	114427899	rs46432555	*I:Hrh1*	6.32E-05
6	114792207	rs31444358	*I:Atg7*	8.71E-05
6	114941577	rs6248634	*NC:LOC105242742*	3.14E-03
6	114951759	rs31456818	*Vgll4*	8.25E-06
6	115008370	rs6262329	*I:Tamm41*	3.14E-03
6	115181005	rs31473897	*I:Syn2*	6.72E-04
6	115286744	rs31484360	*NA*	8.71E-05
6	115321034	rs31484653	*NC:LOC105242744*	1.31E-02
6	115464239	rs51526492	*I:Pparg*	8.71E-05
6	115565855	rs51720018	*I:Tsen2*	3.14E-03
6	115612395	rs31487271	*I:Mkrn2*	5.54E-04
6	115663927	rs31496654	*I:Raf1*	2.79E-02
6	115753510	rs6160517	*I:Tmem40*	3.14E-03
6	115782690	rs31504457	*I:Cand2*	6.72E-04
6	115853377	rs31503770	*U5:Mbd4*	8.08E-03
6	115879245	rs31507517	*I:Ift122*	1.71E-03
6	115976241	rs31518615	*I:Plxnd1*	2.11E-02
6	116120070	rs31525210	*I:Tmcc1*	3.14E-03
6	116214868	rs31533291	*I:Fam21*	6.72E-04
6	116311087	rs31535245	*I:Zfand4*	1.08E-02
6	116402580	rs31537093	*I:March8*	6.72E-04
6	118643222	rs31587804	*I:Cacna1c*	1.32E-02
6	119353404	rs13472337	*U3:Adipor2*	3.86E-02

This list was generated using a stringent cut-off (*p<3.81E-06*) and a moderate cut-off (*p<5.00E-02*) using Efficient Mixed Model Association (EMMA). Only the most significant SNP tagging a gene is shown. The functional location of variants is listed using the following notation: intronic (I), synonymous change (Cs), 3' untranslated region variant (U3), 5' untranslated region variant (U5), not characterized (NC) and no gene (NA).

Supplementary Table3. Details of primers used in this study.

**Microsatellite primers**			
Name	Mbp		
D6Mit17	71.06	Forward	5’-ggcttgccaacaaaactgat-3’
		Reverse	5’-gggttttccccttcaaatgt-3’
D6Mit8	83.66	Forward	5’-tgcacagcagctcattctct-3’
		Reverse	5’-ggaaggaaggagtggggtag-3’
D6Mit65	101.33	Forward	5’-ctccgcaaacatgtgtatatgt-3’
		Reverse	5’-ggactcaaacttgctcactgg-3’
D6Mit149	105.95	Forward	5’-acatgcatgcacaactccat-3’
		Reverse	5’-ttttttgtgggctgcatgta-3’
D6Mit105	107.74	Forward	5’-ctgctccactacttctattcctgg-3’
		Reverse	5’-caaagccttatatattacacctcacc-3’
D6Mit115	116.61	Forward	5’-ccatttaataagtgatccctctgg-3’
		Reverse	5’-tgtcacaccacaatgggc-3’
D6Mit254	125.3	Forward	5’-agtgtccctaggggggtgg-3’
		Reverse	5’-ggggccttagaggtagcaac-3’
**Sequence-specific primers**			
Name	Mbp		
rs36743061	81.89	Forward1	5’-ctgcagagatgactaacttccacac-3’
		Forward2	5’-ctgcagagatgactaacttccacaa-3’
		Reverse	5’-gacggggcagcaaatcttat-3’
rs30936839	89.22	Forward1	5’-tttatggagagcagcatggag-3’
		Forward2	5’-tttatggagagcagcatggac-3’
		Reverse	5’-tactgctgtggacagccaac-3’
rs29868697	108.45	Forward1	5’-ccacactggtcaggcctc-3’
		Forward2	5’-ccacactggtcaggcctt-3’
		Reverse	5’-attgaaagctcccagcagaa-3’
rs6257334	119.91	Forward1	5’-agttgcttctgttctcattgctatg-3’
		Forward2	5’-agttgcttctgttctcattgctatt-3’
		Reverse	5’-tctagctggggtgcattacc-3’
**Hrh1 primers**			
Name	Mbp		
Hrh1 WT	114.3	Forward	5’-tgaagtatctggctctgagtgg-3’
		Reverse	5’-ccatccgatggctccctccctgggag-3’
Hrh1 KO	114.3	Forward	5’-tgaagtatctggctctgagtgg-3’
		Reverse	5’-tctatcgccttcttgaccgag-3’

Supplementary Table 4. List of datasets retrieved from mouse phenome database (www.phenome.jax.org) to collate SNP information across all studied mouse strains [43, 44, 68-73].

Data set	Procedure	What's in this data set	Panel	Sex	Year
Broad2	genotyping	SNP profiling, 131,000+ genomic locations, 1-19,X.	inbred		2009
CGD-MDA1	genotyping	SNP profiling, 470,000+ genomic locations, 1-19,X,Y,MT.	inbred	m	2014
CGD-MDA2	genotyping	SNP profiling, 470,000+ genomic locations, 1-19,X,Y,MT.	BXD w/par	m	2014
CGD-MDA3	genotyping	SNP profiling, 470,000+ genomic locations, 1-19,X,Y,MT.	ILSXISS w/par	m	2014
CGD-MDA4	genotyping	SNP profiling, 470,000+ genomic locations, 1-19,X,Y,MT.	AXB, BXA, BXH, CXB, AKXL w/par	m	2014
CGD-MDA5	genotyping	SNP profiling, 470,000+ genomic locations, 1-19,X,Y,MT.	B6.A, B6.PWD consomic panels	m	2014
Perlegen2	genotyping	SNP profiling, 8,100,000+ genomic locations, 1-19,X,Y,MT.	inbred	m	2005
Sanger4	genotyping	SNP profiling, 80,000,000+ genomic locations. SNPs and indels. 1-19,X.	inbred	both	2017
UCLA1	genotyping	SNP profiling, 132,000+ genomic locations, 1-19,X	HMDP	both	2018
UNC-GMUGA1	genotyping	SNP profiling, 130,000+ genomic locations, 1-19,X,Y,MT.	CC w/par	m	2020
UNC-MMUGA2	genotyping	SNP profiling, 76,000+ genomic locations, 1-19,X,MT	CC w/par	both	2017

Supplementary Figure 1. Mouse family tree (adapted from Petkov *et al*. [22]) showing the phylogenetic relationships among 102 inbred and wild-derived inbred strains. Group 1, Bagg albino derivatives; Group 2, Swiss mice; Group 3, Japanese and New Zealand inbred strains; Group 4, C57/58 strains; Group 5, Castle mice; Group 6, C.C. Little DBA and related strains; Group 7, wild-derived strains. Strains with $Hrh1^{r}$ allele are highlighted and their Bphs susceptibility color coded. Red = $Bphs^{s}$, Green = $Bphs^{r}$, and Black = Bphs unknown ($Bphs^{?}$).

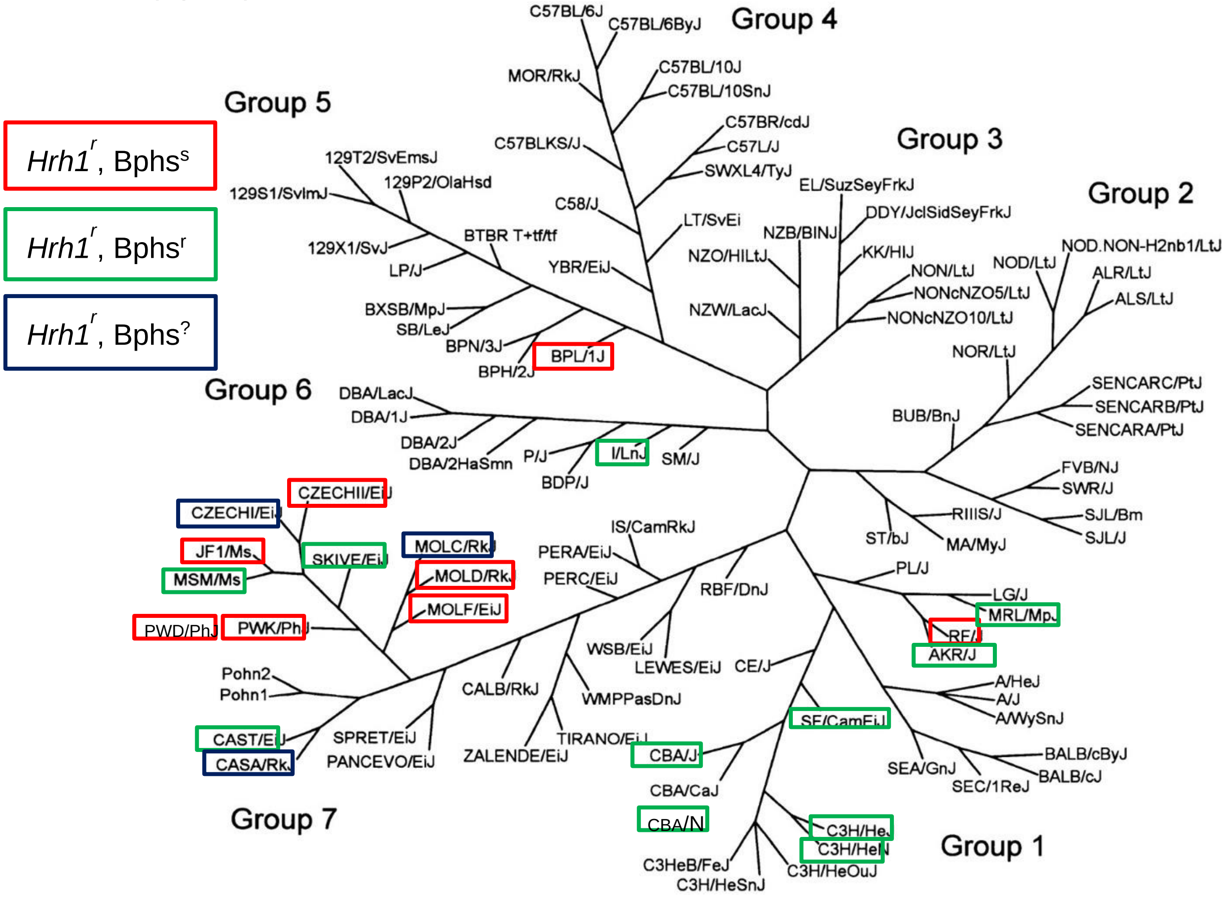

Supplementary Figure 2. The predicted candidates for *Bphse* overlaps with *Bphs/Hrh1*, *Histh3,* and *Histh4* in agreement with the genetic data that a functional LD on Chr6 encodes genes that control both *B. pertussis*/PTX-dependent and age- and inflammation-dependent susceptibility to HA-shock.

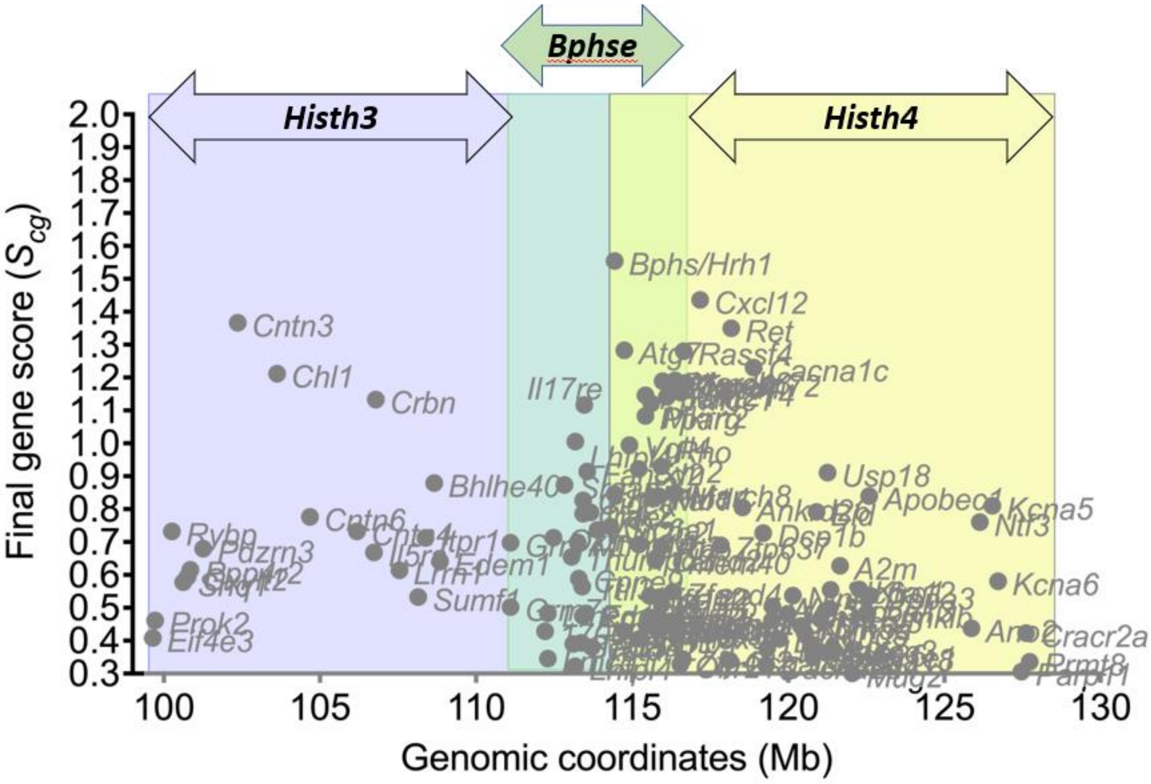

Supplement: Supplementary file 2 — Supplementary Information [file 42003_2023_4603_MOESM2_ESM.pdf]
