# Supplementary material for: A genetic locus complements resistance to Bordetella pertussis-induced histamine sensitization
Source: Commun Biol. 2023 Mar 6;6:244. doi: 10.1038/s42003-023-04603-w (PMC9988836; doi:10.1038/s42003-023-04603-w)
Supplement: Supplementary file 3 — Description of Additional Supplementary Files [file 42003_2023_4603_MOESM3_ESM.pdf]

Description of Additional Supplementary Files

File name: Supplementary Data 1
Description: List of genetic variants identified ($p \leq 0.05$) using imputed genotypes across Chr6:111.0-116.5 Mb.

File name: Supplementary Data 2
Description: List of genes predicted to be functionally associated with Bphs physiological processes ranked by negative log of false positive rate.
