# Supplementary material for: A genetic locus complements resistance to Bordetella pertussis-induced histamine sensitization
Source: Commun Biol. 2023 Mar 6;6:244. doi: 10.1038/s42003-023-04603-w (PMC9988836; doi:10.1038/s42003-023-04603-w)
Supplement: Supplementary file 6 — Reporting Summary [file 42003_2023_4603_MOESM6_ESM.pdf]

nature portfolio

Corresponding author(s): *Double-blind peer review submissions: write DBPR and your manuscript number here instead of author names.*

Last updated by author(s): Feb 2, 2023

Reporting Summary

Nature Portfolio wishes to improve the reproducibility of the work that we publish. This form provides structure for consistency and transparency in reporting. For further information on Nature Portfolio policies, see our Editorial Policies and the Editorial Policy Checklist.

Statistics

For all statistical analyses, confirm that the following items are present in the figure legend, table legend, main text, or Methods section.

n/a	Confirmed	
☐	☒	The exact sample size (n) for each experimental group/condition, given as a discrete number and unit of measurement
☐	☒	A statement on whether measurements were taken from distinct samples or whether the same sample was measured repeatedly
☐	☒	The statistical test(s) used AND whether they are one- or two-sided<br>*Only common tests should be described solely by name; describe more complex techniques in the Methods section.*
☒	☐	A description of all covariates tested
☐	☒	A description of any assumptions or corrections, such as tests of normality and adjustment for multiple comparisons
☒	☐	A full description of the statistical parameters including central tendency (e.g. means) or other basic estimates (e.g. regression coefficient) AND variation (e.g. standard deviation) or associated estimates of uncertainty (e.g. confidence intervals)
☒	☐	For null hypothesis testing, the test statistic (e.g. F, t, r) with confidence intervals, effect sizes, degrees of freedom and P value noted<br>*Give P values as exact values whenever suitable.*
☒	☐	For Bayesian analysis, information on the choice of priors and Markov chain Monte Carlo settings
☒	☐	For hierarchical and complex designs, identification of the appropriate level for tests and full reporting of outcomes
☒	☐	Estimates of effect sizes (e.g. Cohen's d, Pearson's r), indicating how they were calculated

Our web collection on statistics for biologists contains articles on many of the points above.

Software and code

Policy information about availability of computer code

Data collection	Gene Weaver 2.0
Data analysis	SolexaQA version 3.1.7.3, BWA version 0.7.17.tar.bz2, Samtools version 1.16.1, GATK version 4.3.0.0, HaploQA version 1.0, Chromas v2.6.5

For manuscripts utilizing custom algorithms or software that are central to the research but not yet described in published literature, software must be made available to editors and reviewers. We strongly encourage code deposition in a community repository (e.g. GitHub). See the Nature Portfolio guidelines for submitting code & software for further information.

Data

Policy information about availability of data

All manuscripts must include a data availability statement. This statement should provide the following information, where applicable:

- Accession codes, unique identifiers, or web links for publicly available datasets
- A description of any restrictions on data availability
- For clinical datasets or third party data, please ensure that the statement adheres to our policy

All data generated or analysed during this study are included in this published article, Supplementary Information, and Supplementary Data. The Source data underlying the plots shown in Figs. 2–3 are provided in Supplementary Data 2-3. This trait can be accessed under the name Histh. SNP data were retrieved from publicly available databases (mouse phenome database, https://phenome.jax.org/snp/retrievals; Mouse genomes project,https://www.sanger.ac.uk/sanger/

Mouse_SnpViewer/rel-1505). A list of studies that have been used to generate phenotypic data are available at: https://phenome.jax.org/about/snp_retrievals_help.

Human research participants

Policy information about studies involving human research participants and Sex and Gender in Research.

Reporting on sex and gender	n/a
Population characteristics	n/a
Recruitment	n/a
Ethics oversight	n/a

Note that full information on the approval of the study protocol must also be provided in the manuscript.

Field-specific reporting

Please select the one below that is the best fit for your research. If you are not sure, read the appropriate sections before making your selection.

☒ Life sciences ☐ Behavioural & social sciences ☐ Ecological, evolutionary & environmental sciences

For a reference copy of the document with all sections, see nature.com/documents/nr-reporting-summary-flat.pdf

Life sciences study design

All studies must disclose on these points even when the disclosure is negative.

Sample size	Bphs is an extremely penetrant phenotype (100%) as described in Ma etal., 2002 paper. We utilized >3 animals per genotype per sex to study Bphse giving us a fairly confident sample size to study
Data exclusions	n/a
Replication	All experiments were repeated atleast thrice and data pooled to incorporate variability between experiments
Randomization	We tested animals in batches depending on availability from Jax laboratories. Since several of the wild derived strains have difficulty breeding we had to rely on vendor availability so any strain that was made available we tested it.
Blinding	it was not relevant to the study because we had no idea we will find any Hrh1 resistant animals that are susceptible to Bphs. It was serendipity that we came across this group of animals and then we did confirmatory tests to identify the genetic locus responsible for this phenotype.

Reporting for specific materials, systems and methods

We require information from authors about some types of materials, experimental systems and methods used in many studies. Here, indicate whether each material, system or method listed is relevant to your study. If you are not sure if a list item applies to your research, read the appropriate section before selecting a response.

Materials & experimental systems

n/a	Involved in the study	
☒	☐	Antibodies
☒	☐	Eukaryotic cell lines
☒	☐	Palaeontology and archaeology
☐	☒	Animals and other organisms
☒	☐	Clinical data
☒	☐	Dual use research of concern

Methods

n/a	Involved in the study	
☒	☐	ChIP-seq
☒	☐	Flow cytometry
☒	☐	MRI-based neuroimaging

Animals and other research organisms

Policy information about studies involving animals; ARRIVE guidelines recommended for reporting animal research, and Sex and Gender in Research

Laboratory animals	AKR/J (AKR), BPL/1J, C3H/HeJ (C3H), C3H/HeN, CAST/EiJ, C57BL/6J (B6), CBA/J (CBA), CBA/N, CZECHII/EiJ, I/LnJ, JF1/MsJ, MOLD/EiJ, A large number of diverse mice strains from Mus musculus (M. m) domesticus (WSB), M. m. musculus (PWK, PWD, CZECHI, and CZECHII), M. m. castaneus (CAST), M. m. molossinus (JF1, MSM, MOLF, MOLD, MOLC), M. m. hortulanus (PANCEVO), M. m. spretus (SPRET), M. m. praetextus (IS), or hybrids of M. m. musculus and M. m. domesticus (SKIVE), M. m. musculus and M. m. poschiavinus (RBF) and of M. m. castaneus and M. m. domesticus (CALB) as well as AKR/J (AKR), BPL/1J, C3H/HeJ (C3H), C3H/HeN, CAST/EiJ, C57BL/6J (B6), CBA/J (CBA), CBA/N, CZECHII/EiJ, I/LnJ, JF1/MsJ, MOLD/EiJ, MOLF/EiJ (MOLF), MRL/MpJ (MRL), MSM/Ms, PWD/PhJ, PWK/PhJ (PWK), RF/J, SF/CamEiJ, and SKIVE/EiJ were purchased from the Jackson Laboratory (Bar Harbor, Maine). The age of animals was between 8-14 weeks and were rested for 2 weeks prior to any experiments.
Wild animals	n/a
Reporting on sex	We utilized animals from both sexes and found no gender differences in susceptibility or resistance to Bphs so we pooled the data
Field-collected samples	n/a
Ethics oversight	All animal studies were approved by the Institutional Animal Care and Use Committee of the University of Vermont

Note that full information on the approval of the study protocol must also be provided in the manuscript.
